# Advances in research on the effects of bile acids and their receptors on intestinal function

**DOI:** 10.3389/fnut.2026.1821418

**Published:** 2026-04-21

**Authors:** Yulin Cheng, Lixia Zhang, Mingrong Zhang, Jiaxiu Yu

**Affiliations:** 1 Sichuan Cancer Hospital and Institute, Chengdu, China; 2 Wuhou District People’s Hospital, Chengdu, China; 3 Wuhou District Health Hospital Woman and Children, Chengdu, China

**Keywords:** bile acid receptors, bile acids, gut microbiota, intestinal homeostasis, therapeutic targets

## Abstract

Bile acids (BAs), once regarded primarily as detergents facilitating lipid digestion, are now recognized as pivotal signaling molecules that orchestrate intestinal and systemic physiology through a diverse network of nuclear and membrane receptors, with distinct receptor classes mediating complementary transcriptional and rapid signaling responses, including Farnesoid X Receptor (FXR), Takeda G protein-coupled Receptor 5 (TGR5), Pregnane X Receptor (PXR), Peroxisome Proliferator-Activated Receptor *α* (PPARα), Vitamin D Receptor (VDR), and Mas-related G protein-coupled Receptor member X4 (MRGPRX4). This review synthesizes recent advances in understanding the molecular architecture of BA signaling, emphasizing receptor structural diversity, spatiotemporal expression patterns along the gastrointestinal tract, ligand specificity shaped by BA chemical modifications, and the emerging roles of microbiota-derived bile acid derivatives and selected non-canonical host targets in intestinal immune and metabolic regulation. Central to this signaling axis is the gut microbiome, which enzymatically reprograms the BAs pool through deconjugation, dehydroxylation, oxidation, and epimerization, as well as emerging reconjugation/amidation pathways, thereby generating classical secondary BAs as well as structurally novel metabolites that modulate host receptor activity and immune-cell programs. In turn, BAs shape microbial composition, establishing a dynamic bidirectional feedback loop critical for maintaining intestinal homeostasis. In addition to classical receptor signaling, selected microbiota-derived BAs metabolites can also influence immune-associated transcriptional regulators, thereby expanding the scope of BAs signaling in mucosal immune homeostasis. Beyond metabolism, BAs-receptor interactions integratively regulate gut barrier integrity via tight junction reinforcement, modulate immune responses through anti-inflammatory pathways and tolerogenic cell induction, and influence gut motility and neuroendocrine signaling. Dysregulation of BAs receptor and metabolite-mediated signaling axes is increasingly implicated in the pathogenesis of inflammatory bowel disease, bile acid malabsorption, diarrhea-predominant irritable bowel syndrome, colorectal cancer—via DNA damage and Wnt/*β*-catenin pathway activation—and systemic conditions such as obesity, non-alcoholic fatty liver disease, and sepsis-related intestinal injury. Emerging therapeutic strategies aim to restore BAs signaling balance through next-generation receptor modulators, tissue-targeted delivery systems, microbiome-directed interventions, rational use of sequestrants, and synergistic combination therapies, thereby supporting the development of more precise and mechanism-based interventions. Future progress will hinge on interdisciplinary approaches integrating metabolomics, gnotobiotic models, and clinical translation to harness the full therapeutic potential of the BAs signaling network in gastrointestinal and metabolic health.

## 1 Introduction

Traditionally viewed as detergents facilitating lipid digestion, bile acids (BAs) are now recognized as central signaling molecules that orchestrate a wide array of intestinal functions through interactions with a diverse network of receptors (1, 2). This paradigm shift has redefined our understanding of BAs biology, positioning them as key regulators of gut barrier integrity, immune homeostasis, motility, and neuroendocrine signaling (3, 4). The gut serves as the primary site where BAs—shaped by both host metabolism and microbial transformation—exert their pleiotropic effects, integrating signals from the liver, microbiota, and enteroendocrine system to maintain physiological equilibrium (5, 6).

Dysregulation of BAs signaling, whether due to altered synthesis, impaired receptor function, or microbiota-driven shifts in the BAs pool, is increasingly implicated in the pathogenesis of gastrointestinal and systemic disorders, including inflammatory bowel disease (IBD), irritable bowel syndrome with diarrhea (IBS-D), colorectal cancer, and metabolic dysfunction (7–10). Notably, disturbances in the balance between primary and secondary BAs—often driven by dysbiosis—can disrupt receptor activation patterns, compromise the function of the intestinal epithelial barrier, and trigger aberrant immune responses (11–13). These insights highlight the clinical importance of BAs microbiota crosstalk. Within this regulatory network, specific measurable entities (e.g., fecal or serum bile acids, C4, FGF19) are candidate diagnostic biomarkers, and pharmacological modulation of this network is a promising therapeutic approach.

This review aims to synthesize recent advances in understanding how BAs and their receptors regulate intestinal physiology and contribute to disease. We first delineate the molecular architecture of BAs receptors—including FXR, TGR5, PXR, VDR, PPARα, and MRGPRX4—as well as their spatiotemporal expression patterns and ligand selectivity along the gastrointestinal tract. We then discuss how commensal microbes reshape the BA pool through deconjugation, dehydroxylation, epimerization, and other biotransformations, thereby creating dynamic feedback loops that influence host signaling. Meantime, the functional section of this review is organized according to receptor classes, with separate discussions of nuclear receptor-mediated regulation and membrane receptor-mediated regulation of intestinal homeostasis. Within this framework, we examine how distinct BA receptor families coordinate barrier integrity, immune tolerance, secretion, motility, and neuroendocrine signaling, and how disruption of these pathways contributes to intestinal and systemic diseases. Finally, we evaluate emerging therapeutic strategies—including receptor-selective modulators, microbiome-directed interventions, and rational use of bile acid sequestrants—with particular emphasis on their potential to restore intestinal homeostasis. By integrating receptor biology, microbial metabolism, and disease relevance, this review aims to provide a more mechanistically grounded perspective on the BAs receptor axis in precision gastroenterology.

## 2 Molecular architecture of bile acid signaling

### 2.1 Structural and functional diversity of BAs receptor

BAs signaling is mediated through a diverse array of nuclear and membrane-bound receptors that collectively enable nuanced physiological responses to fluctuating BAs concentrations and compositions (14, 15). The farnesoid X receptor (FXR/NR1H4), a ligand-activated transcription factor belonging to the nuclear receptor superfamily, remains the best-characterized BAs sensor (16, 17). FXR forms a heterodimer with retinoid X receptor (RXR) and binds to FXR response elements (FXREs) in target gene promoters, regulating BAs synthesis (CYP7A1 repression), transport (upregulation of BSEP, OSTα/*β*), and detoxification (SULT2A1, UGT2B4) (18–20). For instance, recent research has revealed that in mice, liver-specific FGF4 serves as a novel hepatic mediator downstream of FXR and FGFR4, forming an FXR-FGF4-FGFR4 signaling pathway that specifically suppresses the transcription of Cyp8b1 and helps maintain bile acid homeostasis under cholestatic stress (21). Crystallographic studies reveal that FXR’s ligand-binding domain (LBD) accommodates conjugated primary BAs like glycochenodeoxycholic acid (GCDCA) with high affinity (Kd ~ 10–50 μM), inducing conformational changes that facilitate coactivator recruitment (22).

Complementing FXR’s genomic actions, the G protein-coupled bile acid receptor 1 mediates rapid, non-genomic signaling. TGR5 couples primarily to Gαs, activating adenylate cyclase and increasing intracellular cAMP, which in turn stimulates energy expenditure in brown adipose tissue and GLP-1 secretion from enteroendocrine L-cells (23). Unlike FXR, TGR5 exhibits higher affinity for secondary BAs such as lithocholic acid (LCA) and deoxycholic acid (DCA), reflecting its role as a microbial metabolite sensor (24). The human ortholog MRGPRX4, expressed in sensory neurons and cholangiocytes, may contribute to pruritus in cholestatic conditions through Gq-mediated calcium mobilization (25).

Beyond these canonical receptors, BAs modulate several xenobiotic and metabolic sensors. Pregnane X Receptor (PXR), acting as an exogenous sensor, is capable of detecting the presence of foreign toxic substances and maintaining homeostasis within the body by regulating the expression of downstream target genes. It plays a significant regulatory role in the body’s bile acid metabolism and elimination processes (1). Studies have found that rifampicin can activate the interaction between PXR and HNF4α, triggering the release of PGC-1α, thereby inhibiting the transcription of the CYP7A1 gene in human primary hepatocytes (26). The Vitamin D Receptor (VDR) serves as a sensor for intestinal BAs. Although the primary BAs, cholic acid (CA) and chenodeoxycholic acid (CDCA), do not activate VDR, LCA is an effective VDR agonist (27). Nishida et al. (28) found that, compared with wild-type mice, VDR knockout mice had lower levels of total BAs and CDCA in the liver, feces, and urine after being fed a diet containing CDCA; meanwhile, plasma total BAs and LCA levels were relatively higher, indicating that VDR gene deficiency affects CDCA metabolism and that VDR may play a role in the excretion of excess BAs. Therefore, the primary role of VDR in bile acid homeostasis regulation is to promote the detoxification of BAs. Peroxisome proliferator-activated receptor alpha (PPARα) is indirectly regulated by BAs through fibroblast growth factor 19 (FGF19)-mediated suppression of hepatic lipogenesis, linking BAs signaling to lipid metabolism (29). After treating PPARα knockout mice with Wy, it was found that the mRNA expression of FXR did not change significantly, but the mRNA expression of SHP was significantly downregulated, indicating that PPARα may mediate bile acid synthesis by affecting the FXR-SHP pathway. Notably, analysis of the luciferase reporter gene in FXR−/− and PPAR*α*−/− mice revealed that the inhibition of the FXR signaling pathway by PPARα may be due to the competitive utilization of retinoid X receptor α (RXRα). Therefore, PPARα may interact with FXR, thereby inhibiting the regulation of bile acid homeostasis by FXR (30). This structural and functional diversity enables context-dependent responses: for instance, during cholestasis, elevated LCA activates both protective (PXR/VDR-mediated detoxification) and pathological (MRGPRX4-mediated pruritus) pathways, illustrating the dual nature of BAs signaling (31). This receptor multiplicity allows BAs to integrate metabolic, inflammatory, and detoxification pathways across tissues. The following Table 1 summarizes key BAs receptors, their ligands, signaling mechanisms, and physiological roles.

**Table 1:** Major bile acid receptors, their ligands, signaling mechanisms, and physiological characteristics.

Receptor	Primary ligands	Signaling mechanism	Key physiological functions	Tissue distribution	References
FXR (NR1H4)	CDCA > DCA > LCA	Genomic (RXR heterodimer)	BAs homeostasis, glucose/lipid metabolism, anti-inflammation	Liver, ileum, kidney	(21, 22)
TGR5 (GPBAR1)	LCA > DCA > TLCA	Non-genomic (Gαs/cAMP)	Energy expenditure, GLP-1 secretion, immunomodulation	Brown adipose tissue, intestine, immune cells	(23, 24)
PXR (NR1I2)	LCA (high conc.)	Genomic (RXR heterodimer)	Xenobiotic detoxification, BAs clearance	Liver, intestine	(1, 26)
VDR (NR1I1)	LCA (high conc.)	Genomic (RXR heterodimer)	Calcium homeostasis, antimicrobial defense	Intestine, kidney, immune cells	(27, 28)
PPARα	Indirect (via FGF19)	Genomic (RXR heterodimer)	Fatty acid oxidation, anti-steatosis	Liver, skeletal muscle, heart	(29, 30)
MRGPRX4	DCA, LCA	Non-genomic (Gq/Ca^2+^)	Pruritus, cholangiocyte secretion	Sensory neurons, biliary epithelium	(31)

### 2.2 Spatial and temporal dynamics of receptor expression along the gut

The expression of BAs receptors exhibits precise spatial compartmentalization along the gastrointestinal tract, aligning with regional BA concentrations and microbial activity. In the proximal small intestine (duodenum and jejunum), where conjugated primary BAs predominate, FXR expression is relatively low, while ASBT (apical sodium-dependent BA transporter) is absent, limiting BAs uptake (32). FXR expression peaks in the distal ileum—the primary site of BA reabsorption—where it orchestrates the enterohepatic feedback loop via FGF19 secretion (33). Single-cell RNA sequencing of human ileal biopsies confirms that FXR is highly enriched in enterocytes, particularly those expressing ASBT, whereas TGR5 is co-expressed in L-cells and macrophages within the lamina propria (34).

In the colon, where microbial deconjugation and dehydroxylation generate secondary BAs, TGR5 becomes the dominant receptor. Colonic epithelial cells, dendritic cells, and group 3 innate lymphoid cells (ILC3s) express TGR5, enabling BA-mediated regulation of barrier integrity, IL-22 production, and mucosal immunity (35). Notably, TGR5 activation in colonic macrophages suppresses NLRP3 inflammasome activity, reducing IL-1β release—a mechanism relevant to IBD pathogenesis (36). The spatial gradient of receptor expression is further refined by circadian rhythms: hepatic FXR expression oscillates diurnally under control of the core clock gene Bmal1, synchronizing BA synthesis with feeding cycles (37).

Temporal dynamics also manifest during development and disease. In neonates, intestinal FXR expression is low, correlating with immature BA recycling and reliance on maternal BAs pools (38). In contrast, chronic inflammation in IBD downregulates ileal FXR and ASBT, contributing to BAs malabsorption and diarrhea (39). Germ-free mice exhibit blunted colonic TGR5 expression, which is restored upon colonization with 7α-dehydroxylating bacteria, underscoring microbiota-dependent receptor maturation (40). These spatiotemporal patterns highlight the gut as a segmented signaling landscape where BA-receptor interactions are dynamically tuned to local luminal conditions.

### 2.3 Ligand specificity and allosteric modulation by BAs chemistry

The signaling output of BA receptors is exquisitely sensitive to subtle chemical modifications of the BA scaffold, including hydroxylation pattern, side-chain conjugation, and stereochemistry. FXR exhibits highest affinity for dihydroxy BAs like chenodeoxycholic acid, while trihydroxy cholic acid (CA) is a weaker agonist, and monohydroxy LCA acts as an antagonist at physiological concentrations. Conjugation with glycine or taurine enhances water solubility but reduces affinity for FXR by approximately by ~10-fold, shifting signaling toward membrane receptors like TGR5 (41). Microbial transformations reshape receptor signaling by modifying BA structures. Specifically, the 7α-dehydroxylation of CDCA generates DCA, a potent TGR5 agonist, while the epimerization of CDCA to UDCA produces an FXR antagonist with known cytoprotective properties (42).

The complexity of bile acid signaling is significantly expanded through allosteric modulation and ligand selectivity at multiple levels. For instance, synthetic FXR agonists such as obeticholic acid (OCA) can bind to allosteric pockets within the LBD, stabilizing distinct coactivator interfaces by enhancing specific dynamic coupling between helix 5 and helix 7, thereby generating gene expression profiles different from those induced by endogenous BAs (43). Similarly, biased signaling is observed with TGR5: the endogenous ligand LCA primarily induces sustained cAMP production, whereas synthetic agonists like INT-777 have been reported to preferentially activate downstream pathways such as ERK1/2, which may potentially influence different physiological outputs related to cell proliferation versus metabolic responses (44).

Recent studies have considerably expanded the conventional bile acid repertoire by identifying multiple microbiota-derived bile acid derivatives with defined host targets and signaling functions. In addition to classical glycine- and taurine-conjugated bile acids, gut microbes generate microbially conjugated bile salts (MBSCs), including leucocholic acid (LeuCA), phenylalanocholic acid (PheCA), tyrosocholic acid (TyrCA), and their corresponding chenodeoxycholic acid conjugates (45). These metabolites are detectable in human bile, are substrates of the intestinal and hepatic bile salt transporters ASBT and NTCP, and activate both TGR5 and FXR *in vitro*, suggesting that microbial conjugation expands the ligand spectrum of classical bile acid receptors, although these compounds may act predominantly in the intestinal or enterohepatic microenvironment because their entry into the portal circulation appears limited. In parallel, bacterial bile acid amidates (BBAAs), such as glutamido-cholic acid (Glu-CA) and glutamido-chenodeoxycholic acid (Glu-CDCA), further broaden this signaling landscape. These bacterially conjugated bile acids activate several host transcription factors, including FXR, CAR3, PXR, AHR, and PPARα, and induce receptor-responsive target genes in mouse and human small-intestinal organoids, indicating that microbial reconjugation can substantially diversify host bile acid sensing beyond the canonical FXR-TGR5 axis (46).

Among microbial amino-acid-conjugated bile acids, tryptophan-conjugated cholic acid (Trp-CA) provides the strongest current evidence for direct metabolic relevance. Trp-CA has been identified as an endogenous ligand of the orphan G protein-coupled receptor MRGPRE, and its activation promotes GLP-1 secretion through both Gs-cAMP and *β*-arrestin-1-ALDOA signaling pathways, thereby improving glucose tolerance in diabetic mice. Importantly, Trp-CA production was linked to bacterial bile salt hydrolase/transferase activity in *Bifidobacterium*, supporting the concept that microbial enzymatic diversification of bile acids can generate functionally important host-signaling molecules rather than passive metabolic by-products (22). Together, these findings indicate that newly identified microbial bile acid conjugates should be considered an emerging layer of bile acid-mediated intestinal and metabolic regulation.

Emerging oxidized and epimerized secondary bile acid derivatives also illustrate how subtle microbial tailoring redirects host signaling toward immune regulation. The lithocholic acid derivative 3-oxoLCA directly binds the transcription factor RORγt and suppresses Th17-cell differentiation, whereas isoalloLCA promotes regulatory T-cell differentiation through a mechanism involving mitochondrial ROS, enhanced Foxp3 expression, and a requirement for the nuclear receptor NR4A1 (47). Another microbial bile acid derivative, isoDCA, acts indirectly on dendritic cells to reduce their immunostimulatory phenotype and promote peripheral Treg generation; notably, FXR ablation in dendritic cells phenocopies important aspects of isoDCA exposure, supporting a functional linkage between this metabolite and FXR-centered tolerogenic programs (48). In addition to altering the ligand selectivity of classical bile acid receptors, microbial remodeling of the bile acid scaffold can also generate metabolites that influence non-canonical immune targets and transcriptional programs. This expansion is mechanistically important because bile acid signaling is not restricted to FXR-, TGR5-, PXR-, or VDR-centered pathways. For example, specific oxidized and epimerized derivatives of lithocholic acid can modulate T-cell differentiation by acting on immune-associated transcriptional regulators, whereas other bile acid species regulate intestinal immune outputs through transcription-factor-dependent programs in innate lymphoid cells (49). These findings indicate that changes in bile acid chemistry reshape not only receptor engagement but also downstream immune-cell fate decisions, thereby broadening the functional scope of bile acid signaling in intestinal homeostasis.

## 3 Microbial reprogramming of the bile acid pool and bidirectional feedback

### 3.1 Enzymatic transformation of BAs by commensal bacteria

The human gut microbiota plays a pivotal role in reshaping the primary BAs pool through a series of enzymatic reactions that profoundly alter BAs chemistry, receptor affinity, and physiological function. Primary BAs-CA and CDCA-are synthesized in hepatocytes from cholesterol and conjugated with glycine or taurine before secretion into the duodenum. Upon reaching the distal ileum and colon, these conjugated BAs encounter a dense consortium of anaerobic bacteria, primarily from the phyla *Firmicutes* and *Bacteroidetes*, which express specialized enzymes capable of deconjugation, dehydroxylation, epimerization, and oxidation (50). The first critical step is deconjugation, mediated by bile salt hydrolases (BSHs), which are widely distributed among genera such as *Lactobacillus*, *Bifidobacterium*, *Clostridium*, and *Bacteroides*. BSH hydrolyzes the amide bonds (the bond between an amino acid and a bile acid) in conjugated bile acids, yielding free (unconjugated) BAs that are less efficiently reabsorbed and more susceptible to further microbial modification (51).

Following deconjugation, a subset of *Clostridium* cluster XIVa and XI species—most notably *Clostridium scindens*—express the bile acid-inducible operon, which encodes a multi-enzyme pathway for 7α-dehydroxylation. This process converts CDCA to LCA and CA to DCA, both of which are classified as secondary BAs. These transformations significantly increase hydrophobicity and membrane permeability, enhancing their detergent-like antimicrobial properties while also altering their signaling profiles (52). Recent *ex vivo* studies employing physiologically relevant human fecal microbiota models have delineated the kinetic profile of this biotransformation, revealing saturable Michaelis–Menten kinetics and a sequential two-step pathway. In this pathway, (CDCA) is first oxidized to 3-oxo-CDCA, followed by reduction to iso- or allo-bile acid intermediates, culminating in 7α-dehydroxylation to yield hyocholic acid (HCA) or hyodeoxycholic acid (HDCA). These microbially generated metabolites, which are detectable in human circulation and feces, exhibit distinct receptor selectivity profiles—such as partial FXR antagonism and PXR activation—suggesting they possess regulatory functions that extend beyond those of classical secondary BAs (53).

In addition to deconjugation, dehydroxylation, oxidation, and epimerization, recent studies have identified microbial reconjugation/amidation as an additional mechanism that expands bile acid chemical diversity. Bile salt hydrolases (BSHs), classically regarded as deconjugating enzymes, have now been shown to possess amine N-acyltransferase activity and to generate bacterial bile acid amidates (BBAAs) from host-derived bile acids (46). This discovery provides an enzymatic basis for the formation of multiple non-classical bile acid conjugates, including glutamate-conjugated species and related microbial amino-acid-conjugated bile acids. Importantly, these newly generated molecules are not chemically inert: several have been shown to activate host receptors and transcription factors, including FXR, PXR, AHR, and related ligand-responsive pathways in intestinal models (47). At the same time, sequential oxidation and epimerization of lithocholic acid and deoxycholic acid yield immune-regulatory derivatives such as 3-oxoLCA, isoalloLCA, and isoDCA, which influence Th17/Treg balance either by directly targeting transcription factors such as RORγt and NR4A1 or by reprogramming dendritic-cell function (49). These findings indicate that gut bacteria do not simply reduce or detoxify bile acids, but actively reassemble the bile acid pool into a broader signaling library with direct relevance to intestinal immune and metabolic homeostasis.

### 3.2 Dysbiosis-induced alterations in secondary BA production

Disturbances to the gut microbial ecosystem—induced by factors such as antibiotics, inflammation, or dietary changes—profoundly disrupt the biosynthesis of secondary BAs (BAs), with direct consequences for host metabolic and immune homeostasis. Broad-spectrum antibiotic treatments, particularly those targeting anaerobic bacteria, drastically deplete key 7α-dehydroxylating taxa such as *Clostridia*. This depletion leads to a marked reduction in systemic and fecal levels of DCA and LCA. In preclinical models, this reduced level of secondary BAs is associated with diminished TGR5-mediated secretion of glucagon-like peptide-1 (GLP-1), reduced whole-body energy expenditure, and aggravated hepatic steatosis, underscoring the metabolic significance of these microbial metabolites (54). Clinically, a dysbiotic signature characterized by reduced abundance of *Clostridium* clusters XIVa and IV and a corresponding shift in the BA pool—featuring decreased fecal DCA and elevated primary BA concentrations—is observed in patients with diarrhea-predominant IBS-D. This altered BAs profile is implicated in disease pathophysiology, potentially driving colonic secretory diarrhea through concurrent overstimulation of the TGR5 receptor and insufficient activation of the FXR (55).

Inflammatory conditions further perturb BA metabolism. In graft-versus-host disease (GVHD), a complication of allogeneic hematopoietic stem cell transplantation, intestinal inflammation selectively depletes secondary BA-producing bacteria, leading to a preferential loss of unconjugated and microbiota-dependent BAs. This collapse in secondary BA pools coincides with increased hepatic FXR activation by residual primary BAs, driving a pro-inflammatory cascade that exacerbates mucosal injury (56). Similarly, in hepatitis B virus (HBV) infection, viral hijacking of BAs transporters alters enterohepatic circulation, indirectly shaping the gut microbiota composition and reducing secondary BAs synthesis, which in turn facilitates viral replication through FXR-dependent mechanisms (57). These findings illustrate a pathogenic loop wherein dysbiosis impairs BAs transformation, and the resulting BA profile further destabilizes microbial ecology.

Quantitative profiling reveals significant disparities in BAs signatures between healthy and dysbiotic states. In healthy individuals, secondary BAs dominate the fecal BAs pool, comprising 70–80% of the total, with DCA and LCA as the predominant species (58). This profile is markedly altered in dysbiosis. For instance, patients with diarrhea-predominant IBS-D exhibit a characteristic shift, with an increased primary-to-secondary BA ratio and higher fecal concentrations of primary BAs like CA and CDCA. The causal role of the microbiota in shaping this profile is demonstrated by interventions such as fecal microbiota transplantation (FMT). In patients with recurrent *Clostridioides difficile* infection, successful FMT rapidly restores the microbial capacity for BA metabolism, leading to a measurable shift from primary to secondary BAs in the gut, which correlates with clinical cure (59). Collectively, these findings position a deficiency in secondary BAs not merely as a correlate but as a functional biomarker of dysbiosis, with direct implications for diagnosing metabolic disturbances and guiding microbiota-targeted therapies.

### 3.3 Bidirectional host-microbiota feedback shaped by BAs

BAs serve as central mediators in a dynamic bidirectional dialogue between the host and its gut microbiota, wherein host-derived BAs shape microbial composition, and microbial-modified BAs, in turn, regulate host gene expression and immune function. Primary BAs, particularly in their conjugated forms, exert potent antimicrobial effects by disrupting bacterial membranes and inducing oxidative stress, thereby constraining the growth of Gram-positive pathogens while favoring bile-tolerant commensals like *Bilophila wadsworthia* and *Clostridium* spp. (60). This selective pressure maintains microbial homeostasis under physiological conditions but can be subverted in disease. For example, high-fat diets increase taurocholic acid secretion, promoting blooms of sulfite-reducing Bilophila, which exacerbate intestinal inflammation through hydrogen sulfide production (58).

Conversely, BAs modified by the gut microbiota function as key signaling molecules that feedback onto host pathways to modulate immunity and metabolism. Secondary BAs, including DCA and LCA, can activate the membrane receptor TGR5 on intestinal immune cells such as macrophages and dendritic cells. This activation suppresses the assembly of the NLRP3 inflammasome and the subsequent release of interleukin-1β (IL-1β), contributing to the maintenance of mucosal immune tolerance. In the liver, the primary bile acid-mediated activation of the nuclear receptor FXR induces the expression of fibroblast growth factor 19 (FGF19). FGF19 then acts on hepatocytes to repress the transcription of CYP7A1, the rate-limiting enzyme in bile acid synthesis, forming a classic negative feedback loop (59). This ileal FXR-FGF19 axis is a prominent regulatory pathway, and its disruption—such as when microbial deconjugation reduces the availability of FXR ligands—can lead to dysregulated bile acid homeostasis. Furthermore, emerging evidence links microbial bile acid metabolism to post-transcriptional gene regulation in the host. Recent epitranscriptomic studies demonstrate that antibiotic-induced gut dysbiosis alters the host N^6^-methyladenosine (m^6^A) RNA methylation profile in tissues like the ileum, a process directly regulated by changes in the bile acid pool composition. This finding establishes a novel mechanistic link between microbial metabolism and the host’s epitranscriptomic regulation (36).

**Figure 1:**
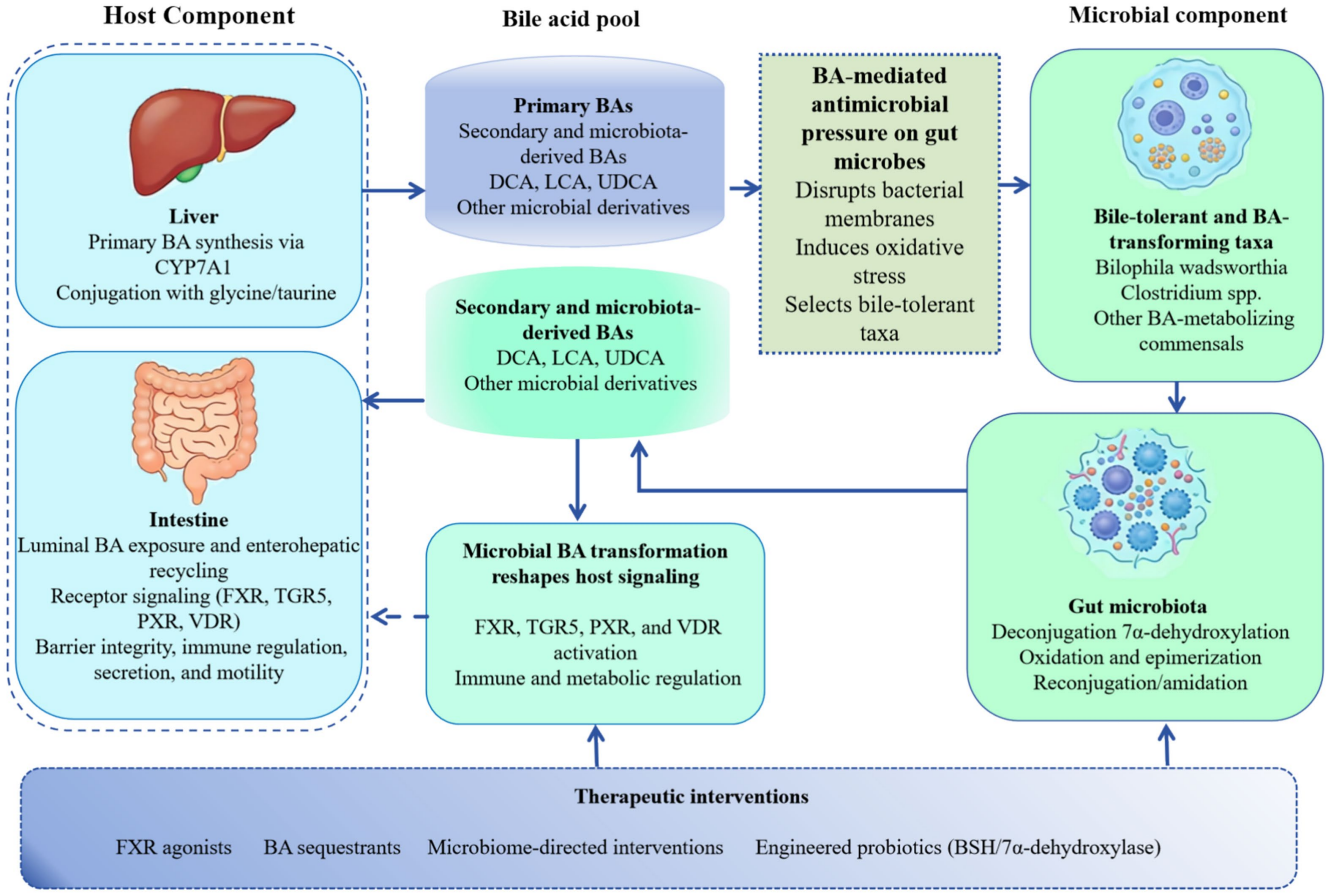
Host-microbiota crosstalk through the bile acid pool: synthesis, microbial transformation, antimicrobial selection, and feedback signaling.

The reciprocal relationship between bile acid and microbiota offers a compelling foundation for therapeutic intervention. A promising strategy involves directly modulating the bile acid pool to steer the gut microbial community toward a healthier configuration. This can be achieved through various means, such as administering pharmacological agents like FXR agonists or bile acid sequestrants, or by utilizing engineered probiotics designed to express key microbial enzymes like bile salt hydrolase (BSH) or 7α-dehydroxylase. These interventions aim to reshape the microbial ecosystem and its metabolic functions (61, 62). Experimental evidence highlights the efficacy of this approach. For instance, treating diet-induced obese mice with glycoursodeoxycholic acid (GUDCA), an endogenous FXR antagonist, has been shown to increase secondary bile acid production. This shift in the bile acid profile leads to a notable enrichment of the beneficial bacterium *Akkermansia muciniphila* and results in improved systemic insulin sensitivity. Observations from physiological states further support the concept of bile acid-driven microbial selection (63). In perinatal sows, natural fluctuations in bile acid profiles correlate with dynamic changes in lipid-metabolizing bacteria, including *Lactobacillus* and *Ruminococcus* (64). This correlation suggests that host bile acid dynamics actively guide microbial community assembly to support major metabolic adaptations, such as those required during gestation and lactation. Collectively, these examples underscore the significant translational potential of targeting the bile acid-microbiota dialogue. By developing interventions that restore this bidirectional equilibrium, novel therapeutic avenues can be opened for treating a spectrum of metabolic, inflammatory, and infectious diseases linked to dysbiosis. Figure 1 illustrates the synthesis, secretion, and signaling pathways of bile acids in the liver and gut, as well as the transformation of bile acids by the microbiota and its own changes.

## 4 Receptor-class-specific regulation of intestinal homeostasis by bile acid signals

BAs regulate intestinal physiology through a coordinated network of receptors that differ not only in cellular localization but also in signaling kinetics, ligand preference, and downstream biological outcomes. In general, nuclear receptors—including FXR, PXR, VDR, and peroxisome proliferator-activated receptor *α* (PPARα)—primarily mediate transcription-dependent programs involved in epithelial protection, BA transport, detoxification, and metabolic adaptation (2, 6, 13). By contrast, membrane-associated receptors—particularly Takeda G protein-coupled receptor 5 (TGR5/GPBAR1) and, to a lesser extent, Mas-related G protein-coupled receptor member X4 (MRGPRX4)—mediate rapid non-genomic responses that influence intestinal secretion, enteroendocrine signaling, immune-cell activation, and motility (35, 37, 45). Although these receptor classes converge functionally in maintaining intestinal homeostasis, distinguishing them mechanistically provides a more coherent framework for understanding how different BA species shape intestinal physiology and pathology.

### 4.1 Nuclear receptor-mediated regulation of intestinal homeostasis

Nuclear BAss receptors provide the major transcriptional platform through which luminal BAss signals are translated into sustained intestinal adaptation. Among them, FXR is the dominant intestinal BAs sensor and plays a central role in coordinating BAs recycling, epithelial defense, and anti-inflammatory signaling. FXR is highly expressed in the terminal ileum, where BAs reabsorption is most active, and is strategically positioned to couple luminal BAs availability to transcriptional programs that preserve mucosal homeostasis (32–34). Upon activation by primary BAss, particularly CDCA, FXR induces the expression of genes involved in BAs transport and efflux, including organic solute transporter *α*/*β* (OSTα/β), while suppressing excessive BAs synthesis through fibroblast growth FGF19-mediated feedBAsck to the liver (18–20, 33). In this way, FXR limits BAs overload within the intestinal lumen and prevents detergent-mediated epithelial injury.

Beyond its role in BAs homeostasis, FXR contributes directly to the preservation of intestinal BAsrrier integrity. Activation of FXR enhances the expression of tight junction-associated proteins such as occludin, claudin-1, and zonula occludens-1 (ZO-1), thereby reducing paracellular permeability and reinforcing epithelial resistance to luminal antigens and pathogens (6, 65). In murine models of dextran sulfate sodium (DSS)-induced colitis, pharmacological FXR activation attenuates epithelial damage, reduces FITC-dextran leakage, and preserves mucosal architecture, whereas FXR deficiency exacerBAstes inflammation and BAsrrier dysfunction (4, 9). These findings indicate that FXR is not merely a metabolic sensor but also a critical guardian of epithelial integrity in the intestine. Recent reviews further support the concept that FXR is a major node linking BAs composition to intestinal epithelial stability and inflammatory restraint.

Other nuclear receptors broaden this transcriptional defense network by sensing hydrophobic or potentially toxic BAs metabolites and inducing detoxification-related programs. PXR is activated by LCA, particularly under conditions of BAs excess, and functions as a xenobiotic-responsive receptor that upregulates genes involved in BAs hydroxylation, sulfation, and efflux (1, 26). Through this mechanism, PXR reduces the intracellular accumulation of cytotoxic BAs species and helps protect the intestinal epithelium from chemical stress. In parallel, VDR also recognizes LCA-derived signals and contributes to BAs detoxification while additionally supporting antimicrobial and immune-regulatory functions in the gut (27, 28). The cooperative activity of FXR, PXR, and VDR therefore establishes a layered nuclear receptor system in which FXR primarily governs physiological BAs homeostasis, whereas PXR and VDR provide adaptive protection against BAs-induced toxicity and inflammatory injury. This distinction is important in intestinal disease states, where dysbiosis and altered BAs composition may reduce the proportion of physiological FXR agonists while increasing exposure to hydrophobic BAs species that require detoxification-driven responses (2, 35, 37).

Compared with FXR, PXR, and VDR, PPARα appears to play a more indirect but still relevant role in BAs-responsive intestinal regulation. Rather than acting as a primary intestinal BAs receptor, PPARα functions at the interface of BAs signaling and lipid metabolic adaptation, partly through crosstalk with the FXR-SHP axis and shared use of RXRα (29, 30). Although the evidence for a direct intestinal role of PPARα in BAs-mediated BAsrrier or immune regulation remains less extensive, its inclusion within the nuclear receptor framework is justified because it contributes to the broader transcriptional landscape through which BAs signals influence nutrient handling, inflammatory tone, and host metabolic status. Collectively, these observations indicate that nuclear receptors mediate a relatively slow but durable layer of intestinal control, centered on gene-expression remodeling, epithelial defense, BAs transport, detoxification, and metabolic adaptation. This receptor class is therefore particularly important for maintaining long-term homeostasis under fluctuating luminal BAs conditions (2, 45).

### 4.2 Membrane receptor-mediated regulation of intestinal homeostasis

In contrast to nuclear receptors, membrane-associated BAs receptors mediate rapid and spatially dynamic responses to changes in luminal and microbiota-modified BAs composition. The best-characterized receptor in this category is TGR5/GPBAsR1, a G protein-coupled receptor that is preferentially activated by secondary BAss such as DCA and LCA (23, 24). Because these ligands accumulate predominantly in the distal intestine and colon following microbial transformation, TGR5 is particularly important for translating microbiota-dependent BAs signals into functional responses in the lower gastrointestinal tract. TGR5 is expressed in multiple intestinal cell populations, including enteroendocrine cells, epithelial cells, macrophages, and other mucosal immune cells, enabling BAs signals to influence secretion, hormone release, immune regulation, and motility in a highly integrated manner (34–36).

One major role of membrane receptor signaling is the regulation of intestinal secretion and fluid BAslance. Through cAMP-dependent pathways, TGR5 activation contributes to epithelial ion transport and chloride secretion, thereby influencing luminal hydration and stool consistency (1, 62). Under physiological conditions, this effect helps maintain intestinal fluid homeostasis. However, when BAs delivery to the colon is excessive—as in bile acid malabsorption (BAsM) or diarrhea-predominant IBS-D—this secretory mechanism becomes pathologically amplified and contributes to diarrhea (1, 62, 66, 67). Secondary BAss are particularly effective in triggering these responses because of their increased hydrophobicity and longer epithelial contact time. Thus, membrane receptor signaling, especially through TGR5, represents a key mechanistic link between altered luminal BAs composition and BAs-driven secretory phenotypes. A broad review of gastrointestinal BAs receptor biology likewise highlights TGR5 as a central mediator of secretion, motility, and enteroendocrine function.

TGR5 signaling also plays a central role in gut neuroendocrine regulation. In enteroendocrine L-cells, activation of TGR5 stimulates intracellular cAMP production and promotes the secretion of glucagon-like GLP-1 and peptide YY (PYY), thereby influencing insulin sensitivity, appetite, and gastrointestinal transit (13, 23). This places TGR5 at the interface between intestinal BAs sensing and systemic metabolic control. In addition, TGR5-mediated signaling has been implicated in enteric neuronal and smooth muscle responses, contributing to the regulation of gastrointestinal motility (63). The rapid, non-genomic character of these membrane receptor pathways distinguishes them from the more transcriptionally oriented functions of nuclear receptors and explains why TGR5 is particularly relevant in conditions characterized by acute changes in luminal BAs exposure, such as post-cholecystectomy diarrhea or BAsM-associated colonic hypersecretion.

Membrane receptor signaling is equally important in the regulation of mucosal immune homeostasis. In lamina propria macrophages and dendritic cells, TGR5 activation suppresses pro-inflammatory signaling pathways, including NLRP3 inflammasome activation, thereby reducing the release of IL-1β and IL-18 (7, 36). This anti-inflammatory effect is especially relevant for secondary BAss such as LCA and DCA, which are generated by microbial metabolism and act as strong TGR5 ligands. Through this mechanism, microbiota-dependent BAs transformation is directly coupled to mucosal immune restraint. Experimental evidence from colitis models further supports this view: loss of TGR5 signaling aggravates intestinal inflammation, whereas TGR5 activation improves BAsrrier integrity and dampens mucosal immune activation (36). A seminal experimental study demonstrated that GPBAsR1/TGR5 modulates intestinal BAsrrier structure and immune responses in colitis, supporting its role as a membrane receptor that links BAs sensing to mucosal protection.

MRGPRX4 is a less well-established but potentially informative addition to the membrane receptor framework. Current evidence primarily links MRGPRX4 to cholestatic pruritus and bile acid sensing in sensory neurons and cholangiocytes rather than to canonical intestinal physiology (25, 31). Nevertheless, its inclusion here is justified because it highlights the expanding scope of non-genomic BAs sensing and suggests that the membrane receptor landscape is broader than TGR5 alone. From the perspective of this review, MRGPRX4 may be best considered an emerging receptor that underscores how elevated or aberrant BAs species can engage additional signaling pathways beyond the classical FXR-TGR5 axis. Taken together, membrane receptors mediate a rapid-response system that converts local BAs fluctuations—especially those driven by microbial metabolism—into coordinated outputs affecting secretion, neuroendocrine function, immune-cell activity, and intestinal motility (2, 23, 24).

### 4.3 Non-canonical bile acid targets and transcriptional regulators in intestinal immune homeostasis

Beyond classical bile acid receptors, several microbiota-derived bile acid metabolites have been shown to influence intestinal immune homeostasis through non-canonical host targets and transcriptional regulators. A representative example is 3-oxoLCA, an oxidized derivative of lithocholic acid, which directly binds the immune transcription factor RORγt and suppresses TH17-cell differentiation (48). This observation was important because it demonstrated that a microbial bile acid metabolite can shape adaptive immunity not only by activating a nuclear receptor or GPCR, but also by directly constraining a lineage-defining transcription factor. In parallel, the related metabolite isoalloLCA promotes regulatory T-cell differentiation by enhancing mitochondrial reactive oxygen species production and increasing FOXP3 expression; subsequent work further showed that the nuclear receptor NR4A1 is required for the full Treg-promoting effect of isoalloLCA (49). Together, these studies establish that bile acid metabolites can directly modulate the transcriptional circuitry governing the BAslance between pro-inflammatory TH17 cells and tolerogenic Treg cells.

Non-canonical immune regulation by bile acid metabolites also extends beyond direct transcription-factor binding. The secondary bile acid derivative isoDCA acts primarily on dendritic cells, where it diminishes their immunostimulatory properties and promotes peripheral Treg generation. Notably, FXR ablation in dendritic cells phenocopies key aspects of isoDCA exposure, suggesting that this metabolite engages a tolerogenic immune program linked to bile acid nuclear receptor signaling but not reducible to the canonical epithelial FXR axis alone (47, 68). These findings support the concept that microbiota-shaped bile acid pools can reprogram immune-cell behavior at multiple levels, including antigen-presenting cell function and downstream T-cell differentiation.

An additional layer of complexity is illustrated by the bile acid-GATA3-ILC3 axis. In a microbiota-dependent metabolic context, glycodeoxycholic acid (GDCA) was shown to stimulate IL-22 production by intestinal group 3 innate lymphoid cells through a GATA3-dependent pathway, thereby improving systemic insulin resistance and disease phenotypes in PCOS-like mice (69). Although this mechanism is distinct from direct ligand binding to transcription factors such as RORγt, it is equally relevant to the present review because it demonstrates that bile acid derivatives can regulate intestinal immunity by modulating transcription-factor-dependent immune programs in innate lymphoid cells. Accordingly, bile acid signaling in the gut should be viewed as extending from classical receptor pharmacology to a broader network of transcriptional control that shapes mucosal immune equilibrium.

### 4.4 Functional convergence and crosstalk between nuclear and membrane receptor pathways

Notably, the intestinal bile acid signaling network is further expanded by emerging microbiota-derived bile acid conjugates and oxidized/epimerized derivatives, some of which engage not only classical bile acid receptors but also immune-facing transcriptional regulators, thereby extending bile acid signaling from epithelial and metabolic control to mucosal immune-cell differentiation (70). Although nuclear and membrane BAs receptors can be distinguished mechanistically, their physiological functions in the intestine are deeply interconnected. In practice, intestinal homeostasis is maintained through the convergence of transcriptional and rapid signaling pathways rather than through the isolated action of individual receptors. For example, microbial conversion of primary BAss into secondary BAss shifts receptor engagement from a profile dominated by FXR toward one that increasingly favors TGR5. This compositional transition affects not only BAs recycling and epithelial gene regulation but also immune-cell signaling, enteroendocrine hormone release, and fluid secretion (35, 37, 42). In this sense, the gut microbiota functions as a biochemical switchboard that redistributes BAs signaling across receptor classes.

BAsrrier protection provides a representative example of such crosstalk. FXR promotes epithelial integrity through transcriptional maintenance of tight junction and transport programs, whereas TGR5 indirectly preserves BAsrrier function by limiting inflammatory injury and restraining innate immune activation (6, 7, 36). Similarly, the BAslance between BAs detoxification and physiological signaling requires coordinated input from FXR, PXR, and VDR at the nuclear level together with TGR5-mediated cellular responsiveness at the membrane level. When this integrated receptor network is disrupted—whether by dysbiosis, inflammation, altered BAs synthesis, or impaired enterohepatic circulation—the result is not a defect in a single pathway but a broader collapse of epithelial, immune, secretory, and neuroendocrine equilibrium (2, 37, 45).

Therefore, a receptor-class-BAssed framework does not fragment intestinal BAs biology; rather, it clarifies how distinct receptor families contribute complementary layers of regulation. Nuclear receptors provide durable transcriptional control over BAsrrier maintenance, BAs transport, and detoxification, while membrane receptors generate fast adaptive responses affecting secretion, hormone release, immune modulation, and motility. Their functional convergence explains why BAs signaling disturBAsnces can manifest simultaneously as BAsrrier injury, chronic inflammation, diarrhea, motility dysfunction, and metabolic dysregulation. This mechanistic integration also provides the conceptual BAssis for the disease-specific disturBAsnces discussed in the following section. The principal bile acid-receptor/host target-intestinal function relationships discussed in this review are summarized in Table 2.

**Table 2:** Major bile acids and microbiota-derived bile acid metabolites, their principal receptors/host targets, and key downstream intestinal functions.

Bile acid/metabolite	Principal receptor(s)/host target(s)	Major downstream intestinal functions	References
CA (cholic acid)	FXR (weak agonist relative to CDCA); indirectly contributes to FXR-FGF19 signaling	Maintains bile acid homeostasis; contributes to epithelial barrier support under physiological conditions; excess colonic exposure promotes secretion and diarrhea	(27, 41, 54, 62)
CDCA (chenodeoxycholic acid)	FXR (major endogenous agonist)	Induces ileal FXR–FGF19 feedback; regulates BA transport and synthesis; supports tight junction expression and barrier integrity; contributes to anti-inflammatory signaling	(18–20, 33, 36, 41)
DCA (deoxycholic acid)	TGR5/GPBAR1; FXR (weaker than CDCA); MRGPRX4 (reported bile acid ligand class)	Stimulates GLP-1 secretion; modulates macrophage inflammasome signaling and mucosal immune tone; promotes chloride/fluid secretion and motility; excessive exposure contributes to secretory diarrhea and colorectal tumor-promoting stress	(23, 24, 34–36, 62, 63)
LCA (lithocholic acid)	TGR5/GPBAR1; PXR; VDR; MRGPRX4	Potent TGR5-mediated immunomodulation and GLP-1 signaling; activates detoxification pathways through PXR/VDR; links microbial BA metabolism to immune restraint; cholestatic bile acid sensor in MRGPRX4-related itch biology	(1, 23, 24, 26–28, 36)
UDCA (ursodeoxycholic acid)	FXR antagonist/weak modulator; TGR5-related motility signaling in some contexts	Cytoprotective effects; modulates motility; may counterbalance excessive FXR activation; contributes to mucosal protection in selected contexts	(42, 63)
GUDCA (glycoursodeoxycholic acid)	FXR antagonist	Reshapes gut microbiota–BAs signaling; associated with improved insulin sensitivity and metabolic homeostasis in obesity-related settings	(59, 106)
Microbially conjugated bile salts (e.g., LeuCA, PheCA, TyrCA; LeuCDCA, PheCDCA, TyrCDCA)	TGR5/GPBAR1, FXR; transported by ASBT and NTCP	Expand the ligand spectrum of classical BA receptors; likely act mainly within the intestinal/enterohepatic microenvironment; potentially influence local metabolic and epithelial signaling	(83, 107)
Trp-CA (tryptophan-conjugated cholic acid)	MRGPRE	Promotes GLP-1 secretion; improves glucose homeostasis; exemplifies direct metabolic signaling by microbial amino-acid-conjugated bile acids	(45, 108)
Bacterial bile acid amidates (e.g., Glu-CA, Glu-CDCA and related BBAAs)	PXR, AhR; broader activation reported for FXR, CAR3, PPARα in screening systems	Extend host sensing beyond canonical BA receptors; induce ligand-responsive transcriptional programs in intestinal models; may influence intestinal immune and metabolic regulation	(46)
GDCA	GATA3-dependent ILC3 pathway	Induces IL-22 secretion; supports mucosal immune and metabolic homeostasis	(69)
3-oxoLCA	RORγt	Inhibits Th17 differentiation; restrains pro-inflammatory intestinal immunity	(48)
isoalloLCA	NR4A1; Foxp3-associated Treg program	Promotes Treg differentiation and immune tolerance	(48, 109)
isoDCA	Dendritic-cell tolerogenic program; FXR-related pathway	Enhances Foxp3 induction and peripheral Treg generation	(48, 49)
3-oxoLCA	RORγt (non-canonical host target)	Suppresses Th17 differentiation; links microbiota-derived BA oxidation to intestinal immune regulation	(23, 24, 48)
isoalloLCA	NR4A1-dependent Treg program; Foxp3-associated regulatory pathway	Promotes Treg differentiation and anti-inflammatory immune tone in the gut	(36, 48)
isoDCA	Dendritic-cell tolerogenic program linked to FXR	Reduces dendritic-cell immunostimulatory capacity; promotes peripheral/colonic Treg generation; supports mucosal immune tolerance	(41, 47, 54)

## 5 Dysregulation of BAs signaling in gastrointestinal and metabolic diseases

### 5.1 Inflammatory bowel disease

IBD, encompassing Crohn’s disease and ulcerative colitis, is characterized by chronic mucosal inflammation driven by genetic susceptibility, immune dysregulation, and gut microbiota alterations. A growing body of evidence implicates BAs signaling disruption as a central pathophysiological mechanism in IBD. Patients with active IBD exhibit marked disturbances in the enterohepatic circulation of BAs, including reduced ileal reabsorption due to ASBT downregulation, diminished FXR activation, and altered microbial metabolism leading to an imbalance between primary and secondary BAs (71).

The molecular consequences of BAs signaling dysregulation in IBD are multifaceted. FXR deficiency in intestinal epithelial cells leads to decreased expression of tight junction proteins and increased permeability, facilitating bacterial translocation and perpetuating inflammation (39). Concurrently, impaired TGR5 signaling in lamina propria macrophages fails to suppress NLRP3 inflammasome activation, resulting in unchecked IL-1β and IL-18 production (72). Preclinical studies utilizing murine models of colitis have substantiated the therapeutic potential of targeting bile acid receptors, demonstrating that pharmacological activation of FXR or TGR5 can ameliorate key disease pathologies. Administration of the FXR agonist obeticholic acid (OCA) in a mouse model of ulcerative colitis significantly reduced the disease activity index, attenuated colonic shortening, and decreased serum levels of pro-inflammatory cytokines such as IL-1β, IL-6, and TNF-*α*, concomitant with improved histological damage (73). This protective effect is associated with the modulation of specific stress response pathways. A hallmark of bile acid dysregulation in IBD is the deficiency in FXR-FGF19 axis signaling, as evidenced by significantly reduced circulating FGF19 levels in patients with Crohn’s disease compared to healthy individuals (74).

Concurrently, profound alterations in the gut microbiota and its metabolic output are evident. Patients with IBD exhibit a marked decrease in the fecal proportions of secondary BAs, which possess anti-inflammatory properties, coupled with an increase in primary BAs. This shift is mechanistically linked to a depletion of specific bile-acid-transforming bacterial taxa and their enzymatic activities. For instance, the levels of several microbially modified BAs, such as 3-oxo-DCA and iso-allo-LCA, which can act as agonists for receptors like GPBAR1, are selectively reduced in IBD patients (75, 76). Beyond impaired signaling through canonical bile acid receptors, disrupted production of immunoregulatory bile acid metabolites may represent an additional layer of immune dysregulation in intestinal inflammation. Emerging evidence suggests that oxo- and allo-bile acid derivatives with TH17/Treg-modulating properties are reduced in inflammatory settings, and that restoring selected metabolites can ameliorate intestinal inflammation in preclinical models. For example, 3-oxo-DCA has been reported to function as a dual GPBAR1 agonist and RORγt inverse agonist, thereby alleviating colitis through coordinated effects on macrophages and TH17/Treg balance (49). These observations broaden the disease framework from a simple deficiency of FXR or TGR5 signaling to a combined loss of receptor-dependent and metabolite-dependent immune regulation within the bile acid–microbiota axis. The integration of BAs profiling into clinical practice is advocated as a strategy to enable more precise, personalized interventions aimed at restoring the BA-microbiota-immune axis to achieve sustained remission.

### 5.2 Bile acid malabsorption and IBS-D

BAM is increasingly recognized as a major, though frequently underdiagnosed, cause of chronic diarrhea, accounting for a substantial subset of patients with diarrhea-predominant IBS-*D. meta*-analyses indicate that up to 30% of patients meeting the diagnostic criteria for IBS-D have evidence of BAM, which can arise from ileal dysfunction (e.g., Crohn’s disease, resection), cholecystectomy, or be idiopathic in nature (77). The pathophysiology of idiopathic BAM is closely linked to a dysregulation of the ileal-bile acid feedback loop. In healthy individuals, BAs reabsorbed in the terminal ileum activate the nuclear receptor FXR, triggering the release of FGF19. FGF19 travels to the liver to potently suppress the expression of CYP7A1, the rate-limiting enzyme in bile acid synthesis. In BAM, this negative feedback is impaired, leading to compensatory hepatic overproduction of BAs and a consequent increase in their delivery to the colon. Within the colon lumen, these unabsorbed BAs, particularly dihydroxy species like deoxycholic acid, act as potent secretagogues. They induce a secretory state by activating apical chloride channels, including the CFTR, on colonic enterocytes, leading to increased chloride and fluid secretion and accelerated transit (78).

The diagnosis of BAM has been advanced by serum biomarkers. Elevated fasting serum 7α-hydroxy-4-cholesten-3-one (C4), a direct marker of hepatic BA synthesis, shows strong diagnostic correlation with SeHCAT testing and is a practical alternative in regions where SeHCAT is unavailable. Patients with confirmed BAM, typically identified by elevated C4 levels, show a significantly better therapeutic response to bile acid sequestrants like colesevelam compared to non-BAM IBS-D patients (79). Beyond driving secretion, excess colonic BAs also contribute to IBS symptomatology by stimulating sensory-motor pathways. They promote the release of serotonin (5-HT) from enterochromaffin cells, which then activates peristaltic reflexes and visceral sensitivity via 5-HT₃ and 5-HT₄ receptors, contributing to abdominal pain and urgency (80).

Therapeutic strategies are increasingly focusing on the modulation of BAs receptor pathways and metabolism to address the root cause of disorders like bile acid diarrhea (BAD). Targeting the FXR represents a direct pharmacological approach. FXR agonists, such as obeticholic acid, aim to restore the ileal FXR-FGF19 negative feedback loop. In a pilot trial involving patients with BAD, treatment with obeticholic acid successfully increased FGF19 levels, which led to the suppression of hepatic BA synthesis and resulted in a clinically significant reduction in stool weight (81). Beyond synthetic drugs, biotherapeutic strategies are under investigation. For instance, probiotic strains engineered to express BSH can deconjugate primary BAs in the gut. This modification reduces their detergent-like, prosecretory effects and promotes their further microbial conversion into secondary BAs with different signaling properties, offering a potential way to reshape the luminal BA pool toward a less diarrheagenic composition (82). The recognition that a substantial subset of diarrhea-predominant IBS-D is driven by BAD enables a shift toward precision medicine. Robust meta-analyses indicate that approximately 32% of patients meeting criteria for IBS-D have co-existing BAD, as diagnosed by the ^75^SeHCAT test (83). This stratification moves clinical management beyond broad symptomatic control with antidiarrheals. Instead, it allows for targeted therapies—such as BA sequestrants or novel FXR agonists—that directly address the underlying pathophysiology of excessive colonic BA exposure, thereby offering the potential for more effective and mechanism-based relief of chronic secretory diarrhea.

### 5.3 Colorectal carcinogenesis

Emerging evidence positions dysregulated BAs signaling as a key driver of colorectal carcinogenesis through genotoxic stress, chronic inflammation, and oncogenic pathway activation. Secondary BAs, particularly DCA, accumulate in the colonic lumen under conditions of high-fat diet or dysbiosis and exert concentration-dependent effects: at physiological levels, DCA promotes mucosal repair via TGR5; at supraphysiological concentrations, it induces oxidative DNA damage, mitochondrial dysfunction, and genomic instability (84). Moreover, BAs modulate critical oncogenic pathways. DCA activates protein kinase C (PKC) and epidermal growth factor receptor (EGFR), leading to downstream stimulation of *β*-catenin and Wnt signaling—a cornerstone of colorectal cancer (CRC) pathogenesis (85).

Human CRC tissues consistently show downregulated FXR expression, correlating with advanced stage and poor prognosis. Conversely, TGR5 overexpression in CRC cell lines enhances cell proliferation via cAMP–CREB signaling, suggesting context-dependent roles for BAs receptors in tumorigenesis. Epidemiologic and mechanistic data converge to support BAs modulation as a chemopreventive strategy. Microbial interventions that lower DCA-producing bacteria (e.g., *C. scindens*) also attenuate tumor burden in preclinical models (86). Monitoring fecal BAs profiles may thus identify high-risk individuals for targeted prevention.

### 5.4 Systemic metabolic dysfunction

BAs serve as metabolic integrators linking nutrient sensing, energy expenditure, and glucose homeostasis, with their dysregulation contributing centrally to obesity and non-alcoholic fatty liver disease (NAFLD). In obesity, altered gut microbiota composition reduces secondary BAs formation, diminishing TGR5-mediated GLP-1 secretion from L-cells and impairing insulin sensitivity. Concurrently, hepatic FXR activation is blunted, failing to suppress lipogenesis and promote fatty acid oxidation, thereby exacerbating hepatic steatosis (87).

Activation of the bile acid receptor FXR has emerged as a validated therapeutic strategy for reversing multiple facets of metabolic syndrome, with its benefits extending from hepatic histology to systemic metabolism. The landmark FLINT trial demonstrated that treatment with the FXR agonist obeticholic acid (OCA) led to significant reductions in liver fat content and was associated with histologic improvement in patients with nonalcoholic steatohepatitis (NASH) (88). Mechanistically, FXR activation orchestrates a metabolic reprogramming that suppresses lipogenesis and enhances energy expenditure. Beyond direct receptor agonism, modulating bile acid circulation offers another pathway. The bile acid sequestrant colesevelam, approved for improving glycemic control in type 2 diabetes, achieves its benefits through a mechanism believed to involve alterations in the enterohepatic circulation of BAs. These pharmacological successes underscore the centrality of the gut microbiota-bile acid axis in metabolic health. Notably, interventions like FMT from lean donors can transfer a favorable metabolic phenotype, partly by enriching bacterial pathways for secondary bile acid production and improving insulin sensitivity in recipients (89). This collective evidence positions precision modulation of the bile acid pool—through diet, prebiotics, or engineered microbial therapies—as a promising frontier for addressing the root causes of metabolic dysfunction.

## 6 Emerging therapeutic strategies targeting the bile acid pathway

### 6.1 Next-generation receptor-targeted agents

The development of next-generation bile acid receptor modulators represents a paradigm shift in the treatment of gastrointestinal and metabolic disorders, with FXR and TGR5 as central therapeutic targets. FXR agonists, such as obeticholic acid (OCA), have demonstrated robust efficacy in non-alcoholic steatohepatitis (NASH) by suppressing hepatic *de novo* lipogenesis through small heterodimer partner (SHP)-mediated inhibition of sterol regulatory element-binding protein 1c (SREBP-1c), while simultaneously enhancing insulin sensitivity and reducing pro-inflammatory cytokine production. In phase III trials, OCA significantly improved fibrosis by at least one stage in 23.1% of NASH patients versus 11.9% in placebo (*p* < 0.001), validating FXR as a druggable node in metabolic liver disease (90). However, dose-dependent pruritus and LDL-C elevation have spurred the development of non-bile acid FXR agonists like tropifexor and cilofexor, which exhibit improved tolerability profiles while maintaining anti-fibrotic activity.

Concurrently, TGR5 agonism has emerged as a promising strategy for enhancing energy expenditure and glucose homeostasis. Systemic TGR5 activation stimulates glucagon-like GLP-1 secretion from intestinal L-cells, improves insulin sensitivity, and promotes brown adipose tissue thermogenesis via type 2 deiodinase-mediated conversion of thyroxine to triiodothyronine (91). However, early pan-agonists induced gallbladder filling and slowed gastric emptying, limiting clinical utility. To circumvent this, gut-restricted TGR5 agonists such as RDX8940 have been engineered to act locally on enteroendocrine cells without systemic exposure, thereby preserving GLP-1 secretory effects while avoiding off-target complications (92). Clinical translation of these multi-target agents is ongoing, with several candidates in phase II trials for NASH and type 2 diabetes. These innovations underscore a move toward precision modulation of BA signaling, balancing efficacy with safety through tissue selectivity and receptor specificity.

### 6.2 Tissue-specific delivery systems

Targeted delivery platforms have emerged as a promising strategy to overcome the limitations of systemic bile acid receptor activation, such as pruritus from neuronal FXR stimulation or gallbladder side effects mediated by TGR5. The core principle is to maximize therapeutic efficacy at the disease site while minimizing off-target exposure and adverse effects. For hepatic diseases like non-alcoholic steatohepatitis (NASH), nanotechnology enables liver-specific targeting. A 2025 study developed an ultrasound-driven, liver-targeted nanobubble system (Apt-DTP-NBs@RSV@OCA) that co-encapsulated obeticholic acid (OCA) and resveratrol. This system demonstrated high encapsulation efficiency (approximately 90% for OCA and 93% for resveratrol) and a spherical morphology with an average diameter of 165 ± 6.05 nm. In a mouse model of NASH, these nanocarriers achieved enhanced hepatic accumulation, activated the FXR/SHP signaling pathway, and effectively alleviated lipid accumulation, inflammation, and oxidative stress, showcasing a sophisticated liver-targeting approach (93).

Similarly, colonic-targeted delivery leverages pH-dependent or microbiota-triggered release mechanisms to localize BAs modulators to the site of intestinal inflammation in IBD. Eudragit S100-coated capsules, which dissolve only at colonic pH > 7, have been used to deliver FXR agonists directly to inflamed mucosa, restoring barrier integrity without systemic absorption. In dextran sulfate sodium (DSS)-induced colitis mice, colonic-release OCA reduced histologic injury scores by 62% and normalized tight junction protein expression (ZO-1, occludin), whereas oral OCA showed minimal colonic bioavailability (94).

Microbiota-responsive polymers that degrade in the presence of bacterial azoreductases offer an alternative strategy, releasing payloads specifically in dysbiotic regions enriched with pathobionts (5). Another frontier involves lymphatic targeting to modulate immune cell function. Lipid-based nanocarriers incorporating long-chain fatty acids are taken up by enterocytes and trafficked via chylomicrons into mesenteric lymph nodes, where they can deliver TGR5 agonists to dendritic cells and macrophages to suppress NLRP3 inflammasome activation (95). These delivery innovations not only enhance therapeutic index but also enable previously undruggable targets to be engaged safely and effectively.

### 6.3 Microbiome-targeted interventions

Given the pivotal role of gut microbiota in bile acid biotransformation—converting primary BAs into secondary forms with distinct receptor affinities—microbiome-targeted strategies have gained traction as indirect modulators of BAs signaling. FMT from healthy donors restores 7α-dehydroxylating bacterial communities, increasing luminal DCA and LCA, which activate colonic TGR5 and FXR to suppress inflammation and enhance barrier function (8). In a pilot trial of ulcerative colitis, FMT recipients exhibited a 2.3-fold increase in fecal secondary BAs and a 50% clinical remission rate at week 8, correlating strongly with microbial engraftment of BA-transforming taxa (96).

Prebiotics and dietary fibers also shape the BAs microbiota axis. Resistant starch fermentation produces short-chain fatty acids that lower colonic pH, inhibiting bile salt hydrolase (BSH)-negative pathobionts while promoting BSH-positive commensals like *Bifidobacterium*, which deconjugate BAs to facilitate secondary metabolism (97). In IBS-D patients with bile acid malabsorption (BAD), a high-fiber diet increased fecal LCA by 40% and reduced stool frequency by 55%, effects attributable to enhanced microbial BAs transformation (98). Engineered probiotics expressing human BSH or 7*α*-dehydroxylase genes represent a next-generation approach. Phage therapy offers another precision tool to selectively deplete bacteria that disrupt BAs homeostasis. CRISPR-engineered phages targeting *B. wadsworthia* reduced its abundance by 99% in murine colitis, normalized BAs composition, and decreased colonic TNF-α by 70% (5). These microbiome-centric interventions exemplify a shift from direct receptor pharmacology to ecological engineering of the BAs metabolic niche.

### 6.4 Rational use of bile acid sequestrants

BAs, traditionally used for hypercholesterolemia, are being repositioned for rational management of BA-related diarrheal disorders and metabolic conditions based on refined patient stratification. Colesevelam, a non-absorbable polymer that binds intestinal BAs, is particularly effective in IBS-D patients with confirmed BAD, which affects approximately 32% of this population as determined by SeHCAT testing (83). In a randomized controlled trial, colesevelam reduced daily stool frequency from 4.2 to 1.8 in BAD-positive IBS-D patients, with 78% achieving adequate relief versus 22% in placebo (*p* < 0.001) (99).

Beyond symptom control, BAs exert beneficial metabolic effects by interrupting enterohepatic circulation, thereby triggering compensatory hepatic BAs synthesis from cholesterol and activating intestinal FXR-FGF19 signaling. In type 2 diabetes, colesevelam lowers HbA1c by 0.5% independently of lipid changes, likely via enhanced GLP-1 secretion and improved hepatic insulin sensitivity (100). However, indiscriminate use can exacerbate fat-soluble vitamin deficiencies or worsen constipation-predominant IBS, necessitating biomarker-guided application.

New-generation BAs with tailored binding affinities are under development to minimize nutrient malabsorption. For example, SC-435 exhibits selective affinity for dihydroxy BAs over trihydroxy forms, preserving micelle formation for lipid digestion while neutralizing secretagogue BAs (101). By mitigating the historical limitations of broad-spectrum sequestrants, this targeted approach ensures that BAS therapy remains a cornerstone of bile acid diarrhea management, evolving from a nonspecific intervention to a more refined, pathophysiology-informed treatment. This evolution is critical for improving long-term patient adherence and outcomes.

### 6.5 Combination therapies

The multifactorial pathogenesis of BAs associated diseases drives the development of combinatorial therapeutic strategies that simultaneously target receptor signaling and downstream metabolic or inflammatory cascades. In non-alcoholic steatohepatitis (NASH), the combination of an FXR agonist with a GLP-1 receptor agonist represents a synergistic approach to address both hepatic steatosis and systemic insulin resistance (102). In IBD, integrating BAs pathway modulators with conventional immunosuppressants aims to achieve synergistic mucosal healing. Preclinical evidence suggests that FXR activation can downregulate NF-κB signaling, which may potentiate the effect of anti-TNF-*α* antibodies like infliximab (103). Supporting this, a study found that the secondary bile acid 3-oxo-DCA, a dual GPBAR1 (TGR5) agonist and RORγt inverse agonist, whose levels are reduced in IBD, can reverse colitis and skew immune responses towards a regulatory phenotype in murine models (75).

For colorectal cancer prevention in high-risk IBD patients, combining BAs sequestrants with chemopreventive agents mitigates genotoxic BAs exposure while targeting oncogenic pathways. Colesevelam reduces luminal DCA, while aspirin inhibits COX-2–mediated prostaglandin E2 production, jointly suppressing *β*-catenin activation (104). In a multicenter observational study, IBD patients on this regimen exhibited 60% lower incidence of dysplasia over 5 years versus controls (105). These rational combinations exemplify a systems-level approach to BAs pathway modulation, maximizing efficacy while minimizing resistance and toxicity across diverse clinical contexts.

## 7 Conclusion

BAs research has undergone a transformative evolution, redefining BAs from simple digestive detergents to central regulators of intestinal and systemic homeostasis. This regulation is achieved through their interactions with a sophisticated network of nuclear and membrane receptors. This review synthesizes compelling evidence that receptor-mediated BAs signaling—primarily via FXR, TGR5, PXR, and VDR—integrates microbial, metabolic, immune, and neuroendocrine cues to maintain gut barrier integrity, modulate inflammation, regulate motility, and influence energy balance. A cornerstone of this paradigm is the bidirectional crosstalk between host BAs metabolism and the gut microbiota. The microbiota enzymatically modifies the primary BAs pool, generating secondary metabolites with distinct receptor affinities that fine-tune physiological responses in health and disease. Dysregulation of this BAs microbiota-host axis is implicated in the pathogenesis of a spectrum of disorders, including IBD, BAD, diarrhea-predominant IBS-D, CRC, obesity, and NAFLD. In these conditions, imbalances in BAs composition can disrupt epithelial barrier function, promote pro-inflammatory signaling, or drive oncogenic processes through mechanisms such as DNA damage and Wnt/*β*-catenin pathway activation. These mechanistic insights are now driving the development of precision therapeutic strategies. Emerging approaches include next-generation, tissue-selective receptor modulators; microbiome-targeted interventions (e.g., engineered probiotics, fecal microbiota transplantation); the biomarker-guided use of bile acid sequestrants (e.g., based on serum C4 levels); and synergistic combination therapies targeting multiple nodes within the BAs–microbiota–immune network. The future of the field hinges on interdisciplinary integration. Leveraging tools from metabolomics, gnotobiotic models, spatial transcriptomics, and digital health will be crucial to decode individualized BAs signaling signatures. Translating these signatures into dynamic, patient-centered interventions will require a shift from static population-based norms to adaptive, biology-informed clinical frameworks. By doi:ng so, the full therapeutic potential of BAs signaling can be harnessed to restore homeostasis, prevent complications, and achieve sustained remission in complex gastrointestinal and metabolic diseases.
